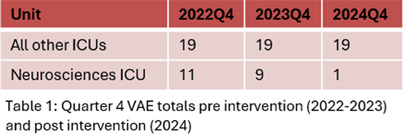# Knobmanship - A Means to Avoid False Positive Burden of VAE

**DOI:** 10.1017/ash.2025.430

**Published:** 2025-09-24

**Authors:** Miranda Neumann, Monica Johnson, Kelly Goetschkes, Lauren Musil, Mark Rupp, Kelly Cawcutt

**Affiliations:** 1 Nebraska Medicine; 2 University of Nebraska Medical Center; 3 Nebraska Medicine; 4 Nebraska Medicine; 5 University of Nebraska Medical Center; 6 University of Nebraska Medical Center

## Abstract

**Background:** Ventilator associated events (VAE) due to changes in positive end expiratory pressure (PEEP) or fraction of inspired oxygen (FiO2) are associated with adverse outcomes for patients in the intensive care unit (ICU). Accurately identifying VAEs is important to improve the quality of care and outcomes for ICU patients. However, we have identified “false-positive” VAEs that are triggered by stylistic manipulation in ventilator settings, or knobmanship, without “true” VAEs that are preceded by signs of hypoxia. This knobmanship creates an undue burden for Infection Preventionists to differentiate clinically relevant VAEs that impact patient outcomes from “false-positive” VAEs. **Methods:** We utilized the Center for Disease Control and Prevention’s National Healthcare Safety Network definition to retrospectively identify VAEs in the pre-pandemic and post-pandemic eras. Of the five ICUs monitored for VAEs, the Neurosciences ICU had the greatest number of events in 2022 and 2023. Working with the NSICU, a pilot study using an initial PEEP of 6 millimeters of mercury (mmHg) rather than 5 mmHg for all intubated patients was conducted. We hypothesized that this would reduce the incidence of “false-positive” VAE without causing adverse patient outcomes. **Results:** Out of 283 reported VAEs from the pre-pandemic period of January 1, 2019, to December 31, 2020, 59 (21%) were due to ventilator changes in PEEP or Fi02 without preceding hypoxia. Post-pandemic evaluation from January 1, 2022, to December 31, 2022, identified “false-positive” VAE in 56 (41%) out of 137 VAE cases. Eighty-two (59%) of the 137 VAE cases were due to changes in PEEP from 5 mmHg to 8 mmHg. After changing the starting PEEP in the NSICU to 6 mmHg from 5 mmHg, from October 1, 2024, to December 1, 2024, only 1 VAE was identified compared to an average of 10 for similar quarters in 2022 and 2023. Despite this change, no adverse events or concerns were noted by the primary ICU team or respiratory therapists. **Discussion:** With thoughtful changes in knobmanship we reduced the burden of “false-positive” VAE without leading to adverse patient outcomes. **Conclusions:** Alteration in starting PEEP can reduce the burden of VAE that are not clinically relevant and allow Infection Preventionists the opportunity to critically analyze clinically relevant VAEs to improve ICU patient outcomes.

**Figure:**